# Phenome-wide association study and functional annotation of hemoglobin A1c-associated variants in African populations

**DOI:** 10.1371/journal.pone.0324269

**Published:** 2025-05-30

**Authors:** Chisom Soremekun, Oyesola Ojewunmi, Amarachukwu Nwagbata, Homa Bazireh, Sapna Sharma, Daudi Jjingo, David Patrick Kateete, Oyekanmi Nash, Harald Grallert, Chiara Batini, Annette Peters, Segun Fatumo

**Affiliations:** 1 NCD Genomics & The African Computational Genomics (TACG) Research Group, MRC/UVRI and LSHTM Uganda Research Unit, Entebbe, Uganda; 2 Department of Immunology and Molecular Biology, School of Biomedical Sciences, Makerere University College of Health Sciences, Kampala, Uganda; 3 Centre for Genomics Research and Innovation, National Biotechnology Development Agency, Abuja, Nigeria; 4 Helmholtz Zentrum München, German Research Center for Environmental Health (GmbH), Institute of Epidemiology, Ingolstädter Landstr, Neuherberg, Germany; 5 Precision Healthcare University Research Institute, Queen Mary University of London, United Kingdom; 6 Usher Institute, University of Edinburgh, Edinburgh, United Kingdom; 7 Faculty of Medicine, Ludwig-Maximilians-University München, München, Germany; 8 African Center of Excellence in Bioinformatics and Data-Intensive Sciences, Infectious Diseases Institute, Kampala, Uganda; 9 Department of Computer Science, Makerere University, Kampala, Uganda; 10 German Center for Diabetes Research (DZD), München-Neuherberg, Ingolstädter Landstr, Neuherberg, Germany; 11 Department of Population Health Sciences, University of Leicester, Leicester, United Kingdom; 12 University Hospitals of Leicester NHS Trust, Leicester, United Kingdom; 13 Institute for Medical Information Processing, Biometry and Epidemiology, Medical Faculty, Ludwig-Maximilians-Universität München, Munich, Germany; University of North Carolina at Chapel Hill, UNITED STATES OF AMERICA

## Abstract

**Background:**

Glycated hemoglobin (HbA1c) measures the average blood sugar level over the past three months. As a vital biomarker of blood glucose levels, it is used to diagnose Type-2 diabetes mellitus (T2D) and monitor glycemic control. A heritability estimate of 47% to 59% suggests that about half of the variation in HbA1c levels can be attributed to genetic factors. Despite African populations being the most genetically diverse and unique for fine-mapping, there is a paucity of data on the genetic drivers of HbA1c in African individuals. In this study, we performed functional annotation and a Phenome-Wide Association Study (PheWAS) of HbA1c-associated variants in two African populations.

**Method:**

In this study, we utilized summary statistics of the HbA1c GWAS meta-analysis of 7,526 individuals from South Africa and Uganda to conduct a PheWAS using GWASATLAS. We also performed a functional analysis using the functional mapping and annotation (FUMA) tool. Single nucleotide polymorphisms (SNPs) were prioritized using the SNP2GENE function, while the gene expression patterns and shared molecular functions were explored in the GENE2FUNC.

**Result:**

Three genome-wide significant loci were identified with the lead SNPs: rs6724428, rs148228241, and rs8045544 – mapped to *GULP1*, *HBA1*, and *ITFG3* genes, respectively. The minor allele frequencies of rs148228241 (0.07) and rs8045544 (0.19) are rare or non-existent in non-African populations. Both rs8045544 and rs148228241 are significantly associated with the mean corpuscular hemoglobin concentration (MCHC). A lower MCHC is associated with alpha thalassaemia, resulting from deletions in *HBA1* and *HBA2* genes. Such deletions are prevalent in malaria-endemic regions of Africa due to their selective survival advantage. The rs6724428 variant is associated with skeletal functions, reflecting the link between glucose metabolism and bone mineral density.

**Discussion:**

Our findings highlight the interplay between glucose metabolism, erythropoiesis, and skeletal health. The significant associations of HbA1c-variants with both skeletal function and MCHC underscore the potential of these variants to impact broader physiological processes. A large-scale study of African individuals will be essential to unravel genetic variants influencing HbA1c.

## Introduction

Glycated hemoglobin (HbA1c) is a measure of the average blood sugar level over the past three months [1]. As a vital biomarker of blood glucose levels, it is used to diagnose Type-2 diabetes mellitus (T2D) and monitor glycemic control [2–4]. According to the American Diabetes Association (ADA), individuals with HbA1c levels between 5.7%–6.4% (39–47 mmol/mol) are prediabetic, while those ≥ 6.5% (48 mmol/mol) indicate T2D [5]. Increased HbA1c levels are linked to cardiovascular diseases and stroke in people with and without diabetes [6].

The first Genome-Wide Association Study (GWAS) of HbA1c levels was conducted on 14,618 women of European ancestry from the Women’s Genome Health Study [7]. Subsequent studies have uncovered more loci associated with HbA1c levels [8,9]. While most GWASs of HbA1c have focused more on Europeans, there is a paucity of data on African individuals. One study has shown that allele frequency and effect size of HbA1c-associated variants are population-specific [10]. Also, African populations have more genetic diversity, which may provide insight into the genetic drivers of complex traits like HbA1c [11]. Hence, it is crucial to investigate the genetic determinants of HbA1c in African populations and to identify the underlying biological pathways and putative functions through fine-mapping and functional annotations.

Phenome-wide association study (PheWAS) is an approach used to uncover the phenotypic associations of genetic variants with other phenotypes [12,13]. Integrating loci identified in GWAS with their biological processes and functions is known as functional annotation of genetic variations [14,15]. Discovering the regulatory elements connected to the variants, patterns of gene expression, and protein functions are all part of these annotations [13,14]. Uncovering relevant biological pathways associated with HbA1c levels will offer insight into possible treatment targets for diabetes management [16,17].

Given the relevance of HbA1c in T2D management and the need to understand its genetic basis in diverse populations, this study aims to investigate the PheWAS and functional implications of HbA1c genetic variation in two African populations.

## Methodology

### Data source

The HbA1c measurement GWAS summary statistics were downloaded from the GWAS catalog with the accession number GCST009054 [18]. This is a meta-analysis of 7,526 individuals from the Uganda Genome Resource (UGR) and the Durban Diabetes Study (DDS) cohort [19,20]. The UGR includes a subset of individuals from the Uganda General Population Cohort (GPC) with available genomic data. The GPC was established in 1989 by the Uganda Virus Research Institute (UVRI) and the Medical Research Council UK (MRC UK) to study the epidemiology of human immunodeficiency virus [21,22]. The UGR comprises SNP array data on ∼5,000 and whole-genome sequence data on ∼2,000 Ugandan GPC individuals from 10 ethno-linguistic groups. The mean age of UGR participants is 30, IQR = 17–46, with females being 56.2%.

The DDS is a population-based study of 1,165 urban black Africans in KwaZulu-Natal, Durban, South Africa, from 2013 to 2014 with mean age 39.7 (95% confidence interval: 38.8–40.7) and 71.5% being females. The study includes genetic information, biophysical measurements, biomarkers for infectious and non-communicable diseases, and the socioeconomic status of study participants [20]. The UGR and DDS association analysis were carried out separately using the linear mixed-model approach implemented in Genome-wide efficient mixed-model analysis (GEMMA v24) [23]. To maximize power for discovery, both datasets were meta-analyzed using the Han-Eskin random effects meta-analysis approach implemented in METASOFT (RE2) [24]. A detailed report on the cohorts, genotyping, quality control, association analysis, and meta-analysis has been reported in our previous paper [19].

### Phenotype measurement

The HbA1c level in both populations was measured using ion-exchange high-performance liquid chromatography certified by the International Federation of Clinical Chemistry and Laboratory Medicine (IFCC) and the National Glycohemoglobin Standardization Program (NGSP).

### Functional annotation and gene expression

Functional analysis was performed with functional mapping and annotation of genetic associations (FUMA) software, which integrates data from several sources using GWAS summary statistics as the input [25]. The embedded SNP2GENE tool was used to map variants to genes and identify independent signals at each locus. Positional mapping (based on ANNOVAR annotations) was done using a maximum distance of 10 kb to map SNPs to genes based on physical distances while including the functional implication of SNPs on genes.

This was followed by the expression quantitative trait locus (eQTL) mapping of SNPs to genes up to 1 Mb part (cis-eQTL) based on eQTL information. Genotype-tissue expression (GTEx) of all available tissues, including whole blood, pancreas, liver, adipose tissue, and skeletal muscle, were used at significant SNP-gene pairs of FDR < 0.05. Only protein-coding genes obtained from Ensembl v102 were included in the gene prioritization. The linkage disequilibrium between SNPs for lead variant selection was obtained using the 1000 Genome Phase 3 African reference panel.

To find pathways enriched for the prioritized genes and explore their expression profiles, we used the GENE2FUNC tool in the FUMA software. Gene-based P-values were obtained using a Multi-marker Analysis of GenoMic Annotation (MAGMA) with its default setting, and the Bonferroni correction was used to correct multiple tests of 19,008 gene sets. This was followed by gene expression heatmap analysis, which illustrated different gene expression levels across several tissues.

### Replication of lead SNPs

To validate the association signals obtained in this study, we used the HbA1c GWAS summary statistics involving African-American individuals from previous studies by Downie et al [26].

### Phenome-wide association study

We performed a PheWAS to investigate the relationship between the HbA1c lead SNPs and other phenotypes. The PheWAS was done using the GWASATLAS, which is a publicly available database with over 4,000 GWAS summary statistics [27]. The rsID of the lead SNPs were imputed in the database, and chromosome and position were extracted from dbSNP (build 146). This was followed by generating a plot of the SNPs across various GWAS in the database. Only SNPs with P-value < 0.05 were included in the plot.

## Results

### Replication of lead SNPs

Three genome-wide significant loci were identified from the meta-analysis: rs148228241 (*P*-value = 2.95x10^-12^) at the *HBA1* locus, rs8045544 (*P*-value = 7.54x10^-09^) at the *ITFG3* locus and rs6724428 (*P*-value = 3.78x10^-09^) at the *GULP1* locus. Using HbA1c GWAS summary statistics obtained from African American individuals [27], we replicated rs148228241 and rs8045544 with the *P*-values 0.006 and 0.008 respectively (Table 1).

**Table 1:** Replication of Genome-wide Significant SNPs associated with HbA_1C_.

			Discovery cohort						Replication cohort
**Gene**	**Lead SNP**	**CHR:BP**	**EA**	**NEA**	**Beta**	**SE**	**EAF**	**P-value**	**P-value**
*GULP1*	rs6724428	2:189377509	A	G	0.085	0.036	0.413	3.788e-09	0.077
*HBA1*	rs148228241	16:227187	G	T	−0.113	0.141	0.074	2.959e-12	0.006
*ITFG3*	rs8045544	16:308002	A	G	−0.120	0.020	0.192	7.540e-09	0.008

EA: Effect allele; NEA: Non-effect allele; CHR:BP (Chromosome:Base pair position - GRCh37); Beta: Beta coefficient; SE: Standard error; EAF: Frequency of the effect allele.

### Functional annotation with FUMA

**Fig 1:**
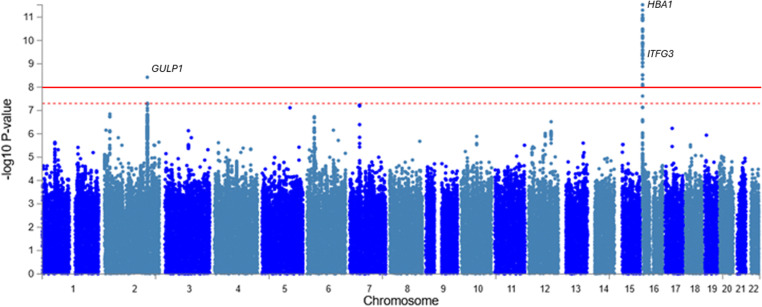
Manhattan plot for the GWAS Meta-analysis of HbA1c trait. Each dot represents a SNP, with its chromosomal position on the x-axis and the -log10 P-value on the y-axis. The red dash horizontal line denotes the suggestive GWAS threshold, and the solid line denotes the actual GWAS threshold (P = 5 × 10 ⁻ ⁸), above which loci are considered statistically significant.

The identified three lead significant SNPs were independent of each other at r^2^ 0.1 (S1 Table). At the genome-wide significant threshold of P-value < 5x10^-8^, eight independent significant SNPs at the r^2^ of 0.1 were identified (S2 Table). 189 candidate SNPs in linkage disequilibrium (LD) with lead SNPs were identified when annotated (Fig 1; S3 Table).

### Functional consequences of SNPs on genes

**Fig 2:**
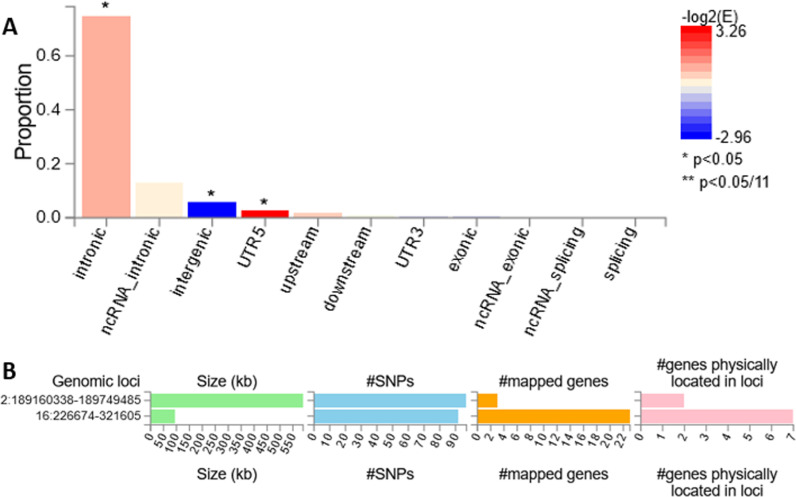
Distribution of SNPs in various functional categories across the genomic regions (A); Summary information per genomic risk locus plot (B).

Most SNPs are found in intronic regions (intronic and ncRNA_intronic), as indicated by the high proportion and significant enrichment (Fig 2A). SNPs in the 5’ untranslated region (UTR5) and upstream regions are less common but show significant enrichment. In contrast, SNPs in downstream, 3’ untranslated region (UTR3), exonic, non-coding RNA Exonic (ncRNA_exonic), non-coding RNA Splicing (ncRNA_splicing), and splicing regions are less common and do not show significant enrichment.

The genomic risk locus plot summary identified two significant loci: one on chromosome 2 (chr2:189160338–189749485) and the other on chromosome 16 (chr16:226674–321605), based on base pair coordinates. SNPs that are dependent on each other at r2 ≥ 0.1 and 500kb are assigned to the same genomic risk locus. The genome-wide significant locus on chromosome 2 is about 600 kb with 100 SNPs and 2 mapped genes, while the locus on chromosome 16 is about 95 kb with 92 SNPs mapped to 7 genes (Fig 2B).

In figure A, the color gradient represents -log2(E) values, where red indicates enrichment and blue indicates depletion. Figure B shows the genomic loci associated with the trait, showing their size (kb), number of SNPs, mapped genes, and physically located genes.

### Gene expression heatmap

#### The heatmap shows the level of expression of some genes in specific tissues.

**Fig 3:**
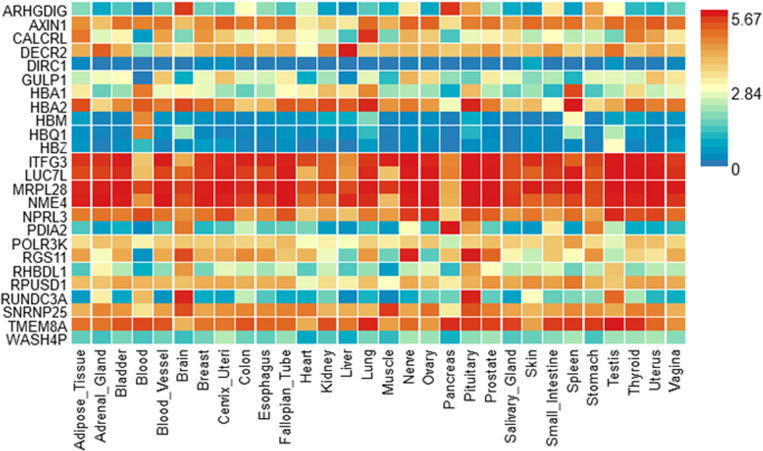
Heatmap comparing gene expression across different tissues. The x-axis represents different tissues, while the y-axis lists the genes analyzed. The color gradient corresponds to the expression levels, with blue indicating low expression, yellow indicating moderate expression, and red representing high expression. The scale bar (0–5.67) represents normalized expression levels.

As expected, *HBA1, HBA2***,** and *HBB* show high expression in blood-related tissues such as the blood and blood vessels*. ITFG3* gene is highly expressed in multiple tissues, including the adipose and reproductive tissues (Fig 3). Examination of the up-regulated and down-regulated differentially expressed genes (DEGs) across GTEx v8 tissue types showed that tissues like blood, spleen, and pancreas show considerable up-regulation. In contrast, tissues like the lungs, thyroid, prostate, and vagina were downregulated. However, the results were not statistically significant after the Bonferroni correction (P_bon_ < 0.05) (S2 Fig).

### Phenome-wide association studies outcome

**Fig 4:**
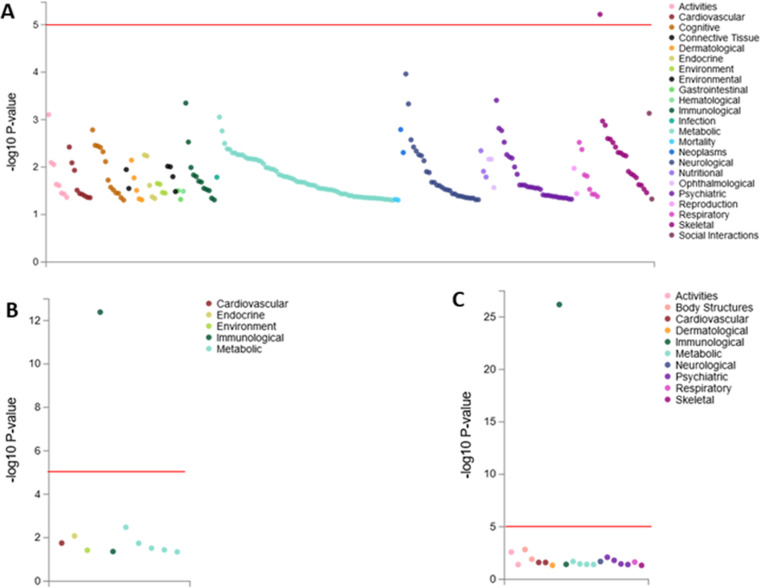
Scatter Plot of the PheWAS Analysis for variants rs6724428 (A), rs148228241 (B), and rs8045544 (C). For each of the plots on the x-axis are different phenotypic traits (e.g., metabolic, cardiovascular, neurological, immunological, etc.), grouped by biological categories and color-coded accordingly, while the y-axis shows the -log10(P-value) of the association between the genetic variant and each trait. Higher values above the red horizontal threshold line are considered statistically significant.

The three lead SNPs were uploaded in the GWASATLAS with 4,756 SNPs in the GWAS summary statistics. Bonferroni’s corrected *P*-value threshold of 1.05x10^-5^ was considered significant. For rs6724428, a plot with 234 data points was generated with Bonferroni’s corrected *P*-value: 2.14x10^-4^. The rs6724428 variant was significantly associated with skeletal functions, precisely the sitting height phenotype (Fig 4A; S4 Table). rs8045544 and rs148228241 have plots with 20 and 10 data points, respectively, and were significantly associated with mean corpuscular hemoglobin concentration (MCHC) with P-values = 6.57x10^-27^ and 4.14x10^-13^, respectively (Fig 4B; S5–S6 Tables).

## Discussion

This study presents the PheWAS and functional annotation of HbA1c-associated variants in two African populations. This is a follow-up to our previous studies, where we examined the genetics of 34 cardiometabolic traits in Ugandans. Our study identified three independent variants associated with HbA1c.

The gene expression level across different tissues helps to explain the gene’s functional roles in the biological processes. Our results show that *HBA1, HBA2*, and *HBB* genes are highly expressed in the blood and blood vessels. The *HBA1* and *HBA2* genes code for the alpha globin proteins, while the *HBB* gene encodes the beta-globin chains of hemoglobin. HbA1c is formed when glucose binds to hemoglobin. Deletion of globin genes impacts the structure and function of hemoglobin, as seen in alpha-thalassemia and sickle cell disease, which may alter HbA1c levels [28].

The PheWAS results show that variants rs8045544 and rs148228241 are significantly associated with the mean corpuscular hemoglobin concentration (MCHC). MCHC is the average concentration of hemoglobin inside a group of red blood cells, and low MCHC is associated with alpha-thalassemia deletion, specifically, α-3·7 kb alpha-globin gene (*HBA1/HBA2*) deletion. This deletion is prevalent in sub-Saharan Africa (33–55%) due to its selective advantage in protecting against severe malaria, and in individuals with sickle cell disease, co-inheritance with alpha-thalasemia is associated with survival [29–31]. Previous studies have established the relationship between HbA1c and MCHC [32–35]. Conditions such as hemolytic anemia can impact hemoglobin synthesis and the lifespan of the red blood cells (RBC), thereby affecting the MCHC and HbA1c levels.

Interestingly, association of rs6724428 with sitting height suggests a potential relationship between metabolic factors and skeletal growth. This trait measures the upper body length, directly reflecting skeletal growth and development [36]. A study by Frayling *et al*. discovered that individuals with shorter stature have a higher risk of T2D [37]. In addition, Wells *et al*. reported the impact of childhood growth patterns on metabolic health and body composition [38]. This can be explained by insulin-like growth factor 1 (IGF-1), a growth hormone that is highly similar to insulin [39]. Insulin is very important in glucose metabolism, and IGF-1 has been reported to play a role in skeletal development and bone remodelling [40,41]. Disruptions in the IGF-1 pathway can lead to compromised skeletal growth (shorter sitting height) and insulin resistance (elevated HbA1c level) [42].

This study has some limitations that may limit the generalizability of our findings. Our study focuses on individuals from two African countries (Uganda and South Africa). Given the African continent’s genomic diversity and demographic complexities, our findings may differ from other African populations. In addition, the sample size of the participants in the meta-analysis might limit the statistical power to detect association signals influencing HbA1c levels. A large-scale GWAS of HbA1c that includes environmental interaction will be essential to uncover genetic variants influencing HbA1c levels. This can improve the power and ensure the findings are representative of the broader African populations.

## Supporting information

S1 Fig Quantile-Quantile (Q-Q) Plot of GWAS P-value. The x-axis represents the expected -log10(P-values) under the null hypothesis, while the y-axis shows the observed -log10(P-values).
(PNG)

S2 Fig Differentially expressed genes across GTEx v8 tissue types. The x-axis represents different tissue types, while the y-axis shows the -log10(P-value) for gene expression significance. Higher values indicate stronger differential expression in the respective tissues.
(PNG)

S1 Table Lead SNPs from 189 genome-wide significant (P-value <5 × 10−8) SNPs. (XLSX)

S2 Table GWAS Metaanalysis Independent Significant SNPs. (XLSX)

S3 Table Annotation of GWAS Metaanalysis Significant SNPs. (XLSX)

S4 Table PheWAS results for the rs6724428 at the GULP1 locus. (XLSX)

S5 Table PheWAS results for the rs8045544 at the ITFG3 locus. (XLSX)

S6 Table PheWAS results for the rs148228241 at the HBA1 locus. (XLSX)
