# Supplementary figures and images for: Phenome-wide association study and functional annotation of hemoglobin A1c-associated variants in African populations
Source: PLoS One. 2025 May 30;20(5):e0324269. doi: 10.1371/journal.pone.0324269 (PMC12124741; doi:10.1371/journal.pone.0324269)

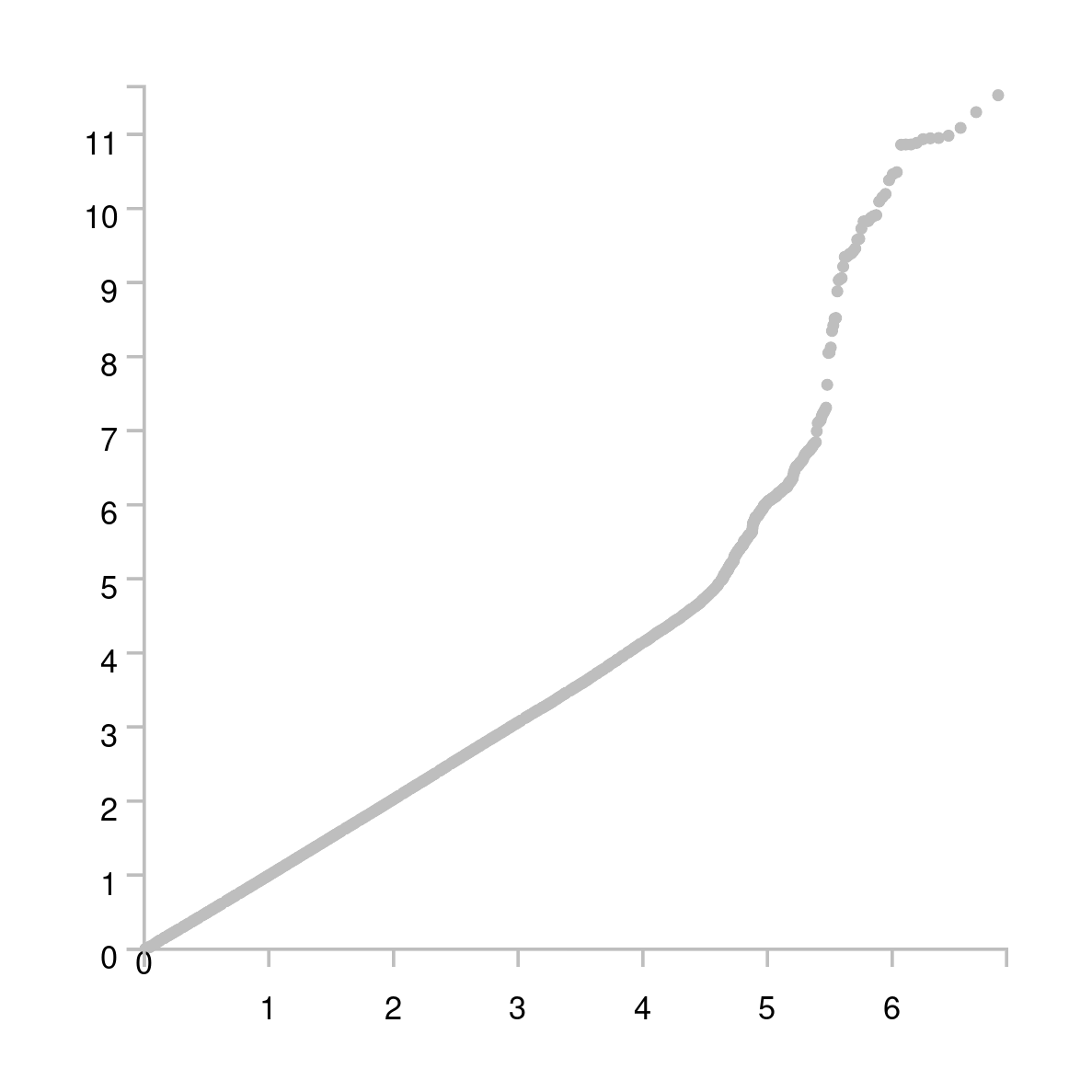

Supplement: S1 Fig — The x-axis represents the expected -log10(P-values) under the null hypothesis, while the y-axis shows the observed -log10(P-values). (PNG) [file pone.0324269.s001.png]

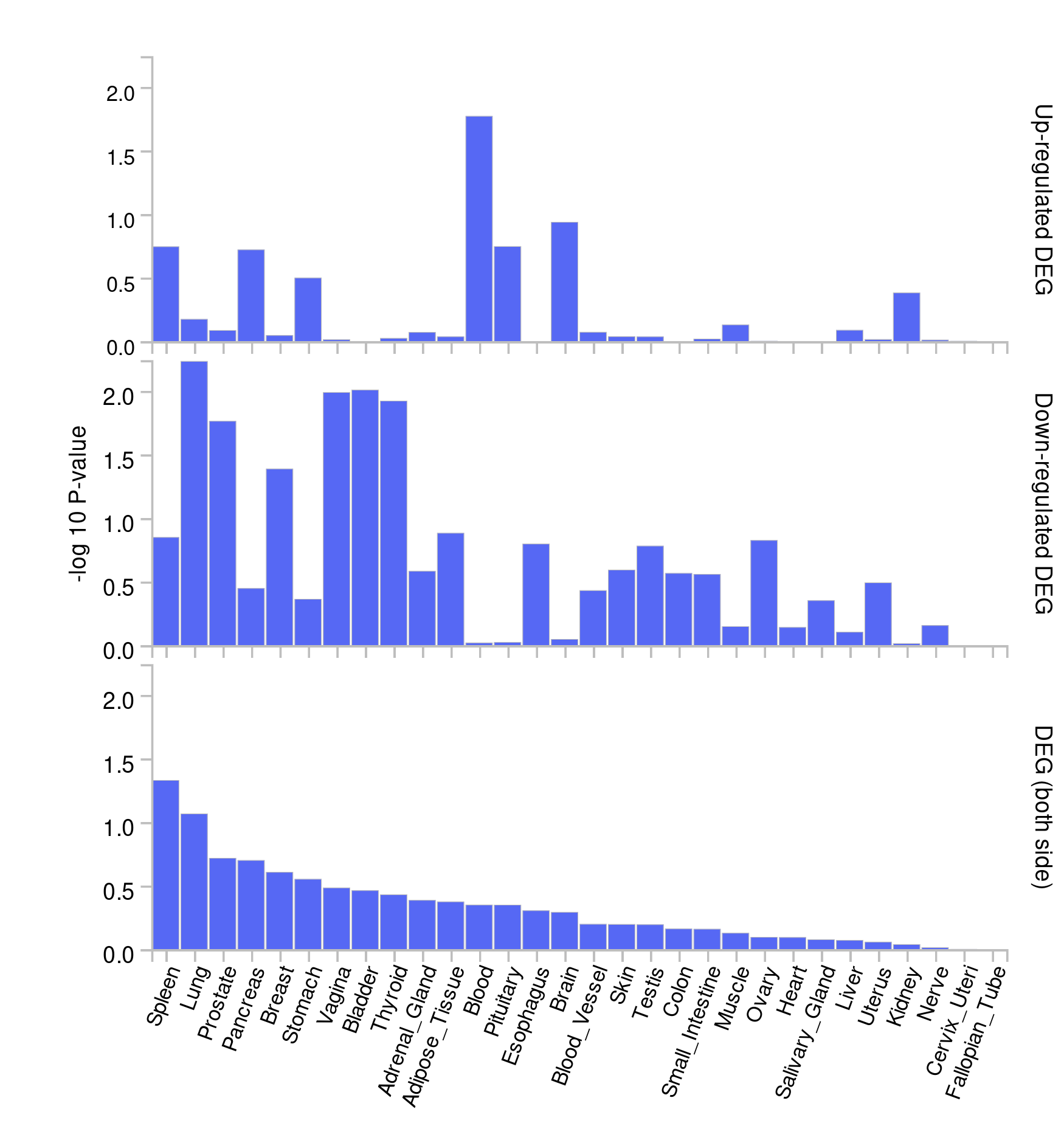

Supplement: S2 Fig — The x-axis represents different tissue types, while the y-axis shows the -log10(P-value) for gene expression significance. Higher values indicate stronger differential expression in the respective tissues. (PNG) [file pone.0324269.s002.png]
